# Inhibition of Osteoblast Function by *Brucella abortus* is Reversed by Dehydroepiandrosterone and Involves ERK1/2 and Estrogen Receptor

**DOI:** 10.3389/fimmu.2018.00088

**Published:** 2018-01-26

**Authors:** María Virginia Gentilini, Ayelén Ivana Pesce Viglietti, Paula Constanza Arriola Benitez, Andrea Elena Iglesias Molli, Gloria Edith Cerrone, Guillermo Hernán Giambartolomei, María Victoria Delpino

**Affiliations:** ^1^Instituto de Inmunología, Genética y Metabolismo (INIGEM), CONICET, Universidad de Buenos Aires, Buenos Aires, Argentina

**Keywords:** *Brucella*, adrenal steroids, immunoendocrinology, cortisol, dehydroepiandrosterone

## Abstract

*Brucella abortus* induces an inflammatory response that stimulates the endocrine system resulting in the secretion of cortisol and dehydroepiandrosterone (DHEA). Osteoarticular brucellosis is the most common presentation of the active disease in humans, and we have previously demonstrated that *B. abortus* infection inhibits osteoblast function. We aimed to evaluate the role of cortisol and DHEA on osteoblast during *B. abortus* infection. *B. abortus* infection induces apoptosis and inhibits osteoblast function. DHEA treatment reversed the effect of *B. abortus* infection on osteoblast by increasing their proliferation, inhibiting osteoblast apoptosis, and reversing the inhibitory effect of *B. abortus* on osteoblast differentiation and function. By contrast, cortisol increased the effect of *B. abortus* infection. Cortisol regulates target genes by binding to the glucocorticoid receptor (GR). *B. abortus* infection inhibited GRα expression. Cell responses to cortisol not only depend on GR expression but also on its intracellular bioavailability, that is, dependent on the activity of the isoenzymes 11β-hydroxysteroid dehydrogenase (HSD) type-1, 11β-HSD2 (which convert cortisone to cortisol and *vice versa*, respectively). Alterations in the expression of these isoenzymes in bone cells are associated with bone loss. *B. abortus* infection increased 11β-HSD1 expression but had no effect on 11β-HSD2. DHEA reversed the inhibitory effect induced by *B. abortus* infection on osteoblast matrix deposition in an estrogen receptor- and ERK1/2-dependent manner. We conclude that DHEA intervention improves osteoblast function during *B. abortus* infection making it a potential candidate to ameliorate the osteoarticular symptoms of brucellosis.

## Introduction

Brucellosis is primarily a disease of domestic and wild animals that can be transmitted to humans, in whom it affects several organs and tissues, given rise to various clinical manifestations. Osteoarticular involvement is the most frequent localization of active disease. Its prevalence varies from one report to another, but a recent study has revealed that as many as 47% of brucellosis patients experienced osteoarticular complications (1). The three most prevalent forms of osteoarticular implications are sacroiliitis, spondylitis, and peripheral arthritis (2–5).

The mechanisms of bone damage due to *Brucella* infection are not completely established. However, we have recently described a putative immune mechanism for inflammatory bone loss that may occur in response to infection by *Brucella abortus* (6). Among many stratagems employed by the bacterium to harm bone, *Brucella* can infect and survive within human and murine osteoblasts, and this infection triggers the secretion of receptor activator of nuclear factor-κB ligand (RANKL), proinflammatory cytokines, and chemokines that could be implicated in the presentation of osteoarticular brucellosis. Such a response is further amplified in the face of *B. abortus* infection by reciprocal influence between osteoblasts and monocytes (7, 8).

Cytokines produced during *Brucella* infection, including those produced during osteoarticular local disease, not only exert a direct effect on immune or bone cells but may also influence this cells indirectly, due to their ability to affect several neuroendocrine mechanisms. Among them, the stimulation of the hypothalamus–pituitary–adrenal axis (HPA) (9). Also, hormones are endogenously released during the course of immune responses. In this context, glucocorticoids and dehydroepiandrosterone (DHEA) may exert important influences on the establishment of the type of immune responses that humans develop against *Brucella* infection. Accordingly, in previous studies it has been demonstrated that cortisol levels were more elevated in patients with acute brucellosis than in healthy individuals (10, 11). In addition, we have previously demonstrated that steroid hormones have a role in the modulation of macrophage response during *B. abortus* infection (11). *Brucella* survives and replicate within vacuolar phagocytic compartments of macrophages (12). This interaction is critical for the establishment of chronic *Brucella* infection; in addition, macrophages have implications in bone disease, not only by the damage induced by the production of proinflammatory cytokines but also by their capacity to differentiate into osteoclast and thus contributing to bone damage due to bone resorption (13).

As mentioned, adrenal steroids not only could modulate immune cell response but could also act on bone cells. Adrenal hormones exert profound effects on bone remodeling (14). However, until now it has not been studied the role of adrenal steroids on bone cells during *B. abortus* infection.

We aimed to determine if the HPA dysregulation observed in acute brucellosis patients evidenced by inappropriately adrenal steroids secretion is implicated in the development and progression of osteoarticular brucellosis. For this, we started by investigating the effects of cortisol and DHEA on osteoblast survival, differentiation, and function during *B. abortus* infection.

## Materials and Methods

### Bacterial Culture

*Brucella abortus* S2308 was grown overnight in 10 ml of tryptic soy broth (Merck, Buenos Aires, Argentina) with constant agitation at 37°C. Bacteria were harvested by centrifugation for 15 min at 6,000 × *g* at 4°C and washed twice in 10 ml of phosphate-buffered saline (PBS). The numbers of bacteria in stationary-phase cultures were determined by comparing the optical densities (OD) at 600 nm with a standard curve obtained in our laboratory. To prepare inocula, cultures were diluted in sterile PBS to the desired bacterial concentration on the basis of the optical density readings, but the precise concentrations of inocula were determined by plating cells onto tryptic soy agar (Britania, Buenos Aires, Argentina). All live *Brucella* manipulations were performed in biosafety level 3 facilities located at the at the Instituto de Investigaciones Biomédicas en Retrovirus y SIDA.

### Cells and Media

The mouse clonal MC3T3-E1 cell line, a standard cell line that has behavior similar to primary calvarial and is useful for studying *in vitro* osteoblast differentiation, was used in the experiments. Unless otherwise specified, all experiments were performed at 37°C in a 5% CO_2_ atmosphere. The cell line were cultured in standard tissue culture flasks containing alpha minimum essential medium (α-MEM), 10% fetal bovine serum, 100 U/ml of penicillin, and 100 g/ml of streptomycin (complete medium). The medium was replaced every 3 or 4 days, and after confluence, cells were harvested using trypsin and resuspended in complete medium.

### Cellular Infection

MC3T3-E1 at a concentration of 3 × 10^5^ cells/well were seeded in 24-well plates or were seeded at 1 × 10^3^ cells/well in 96-well plates (for proliferation assay) and infected at different multiplicities of infection (MOI) in the presence or absence of DHEA (1 × 10^−8^ M) and cortisol (1 × 10^−6^ M), and incubated for 1 h at 37°C in a 5% CO_2_ atmosphere. Cells were extensively washed with α-MEM to remove extracellular bacteria and were incubated in medium supplemented with 100 µg/ml of gentamicin and 50 µg/ml of streptomycin to kill extracellular bacteria in the presence or absence of DHEA and cortisol at the indicated concentrations. MC3T3-E1 cells and culture supernatants were harvested at 24 or 48 h to obtain whole cell extracts and determine chemokines production, matrix metalloproteinases (MMPs) secretion, apoptosis, proliferation, and mRNA extractions.

To monitor *Brucella* intracellular survival, cells were lysed with a sterile solution of 0.1% (vol/vol) Triton X-100 in H_2_O, and serial dilutions of lysates were rapidly plated on tryptic soy agar plates to enumerate colony forming units (CFU).

### Alizarin Red S Staining

To determine calcium deposition, we used alizarin red S staining. On days 7, 14, and 30 of the culture, osteoblasts were fixed in 4% paraformaldehyde for 10 min at room temperature. The cells were washed with deionized water and stained with 2% (wt/vol) alizarin red S and were extracted to perform quantitative analysis by measure the OD at 405 nm.

### Sirius Red Staining

Collagen deposition was quantified by using Sirius red (Sigma-Aldrich, Buenos Aires, Argentina) a strong anionic dye that binds strongly to collagen molecules. Sirius red was dissolved in saturated aqueous picric acid at a concentration of 0.1%. Bouin’s fluid (for cell fixation) was prepared by mixing 15 ml saturated aqueous picric acid with 5 ml 35% formaldehyde and 1 ml glacial acetic acid. Cell layers were extensively washed with PBS before they were fixed with 1 ml Bouin’s fluid for 1 h. The fixation fluid was removed, and the culture plates were washed three times with deionized water. The culture dishes were air dried before adding 1 ml Sirius red dye reagent. The cells were stained for 18 h with mild shaking. The stained cell layers were extensively washed with 0.01 N hydrochloric acid to remove all unbound dye. The stained material was dissolved in 0.2 ml 0.1 N sodium hydroxide by shaking for 30 min. The dye solution was transferred to microtiter plates, and OD measured using a microplate reader (Metertech, Inc., Taiwan) at 550 nm against 0.1 N sodium hydroxide as a blank.

### Signaling Pathway

To study the potential involvement of different signaling pathways in the deposition of organic and mineral matrix by osteoblasts, pharmacological inhibitors [SB203580, a p38 MAPK inhibitor, PD98059, an extracellular signal-regulated kinase 1 and 2 (ERK1/2) MAPK inhibitor, SP600125, a JNK1/2 MAPK inhibitor, and an estrogen receptor (ER) antagonist, fulvestrant] or vehicle [dimethyl sulfoxide (DMSO)] were added at the beginning of culture. Inhibitors (Calbiochem, San Diego, CA, USA) were used at a concentration of 10 µM for MAPK inhibitors and 10 µM for fulvestrant inhibitor, based on previous reports (15–17). Cell viability after incubation with these inhibitors was higher than 90%, as assessed by staining with trypan blue. To account for any possible effect of DMSO on cell viability, cell cultures not treated with the inhibitors were treated with the highest final concentration of DMSO used in these studies (0.01%), and the results were compared with those for cell cultures not exposed to DMSO. In addition, inhibitors do not have a toxic effect on bacterial survival since the levels of invasion and replication were similar to the levels in untreated cells.

### MTT Colorimetric Assay

Cell proliferation/viabiliy was measured by MTT colorimetric assay (Sigma-Aldrich, Argentina) at 48 h postinfection according to the manufacturer’s instructions. The absorbance was measured by using a microplate reader at 570 nm. The OD of each well was quantified as a percentage compared with the untreated osteoblast cells. All experiments were carried out in quadruplicate.

### Bromodeoxyuridine (BrdU) Incorporation Assay

Cell proliferation was examined by using the cell proliferation ELISA BrdU assay (Roche) according to the manufacturer’s instructions. Briefly, at 48 h postinfection MC3T3-E1 were treated with BrdU (10 µM) for 4 h. Then, the cells were fixed and incubated with peroxidase-conjugated anti-BrdU antibody for 90 min. BrdU incorporation was detected by incubating the cells with tetramethyl-benzidine as a substrate. Color development, which was directly proportional to the amount of DNA synthesis and hereby to the number of proliferating cells, was quantified by measuring the absorbance at 370 nm by a microplate reader. The experiments were carried out in triplicate.

### Zymography

Gelatinase activity was assayed by the method of Hibbs et al. with modifications, as described (18, 19).

### Measurement of Keratinocyte Chemoattractant (KC) Concentration

Keratinocyte chemoattractant protein levels in supernatants were determined using the Mouse CXC Chemokine KC DuoSet ELISA Development System (R&D Systems, Minneapolis, MN, USA) according to the manufacturer’s instructions.

### Apoptosis Assays

MC3T3-E1 cell line were infected with *B. abortus* at an MOI of 1,000, and 24 h after infection cells were washed, and the percentage of apoptotic cells was assessed by the annexin V–FITC (Sigma-Aldrich, Argentina) assay with fluorescence-activated cell sorter analysis. The percentage of apoptotic cells was also assessed by fluorescence microscopy after the cells were labeled by the terminal deoxynucleotidyltransferase-mediated dUTP-biotin nick end labeling (TUNEL) assay and by staining with the Hoechst 33342 dye. As a positive control, cells were treated with 200 µM hydrogen peroxide.

### mRNA Preparation and Quantitative PCR

RNA was extracted using the Quick-RNA MiniPrepKit (Zymo Research), and 1 µg of RNA was subjected to reverse transcription using Improm-II Reverse Transcriptase (Promega). PCR analysis was performed with Mx3000P real-time PCR detection system (Stratagene) using SYBR Green as fluorescent DNA binding dye. The primer sets used for amplification were as follows: β-actin sense: 5′-AACAGTCCGCCTAGAAGCAC-3′, β-actin antisense: 5′-CGTTGACATCCGTAAAGACC-3′; RANKL sense: 5′- CTATGATGGAAGGCTCATGG-3′, RANKL antisense 5′-GAGGACAGAGTGACTTTATGG-3′; osteoprotegerin (OPG) sense: 5′-AAGTGTGGAATAGATGTCACC-3′, OPG antisense: 5′-GTATAATCTTGGTAGGAACAGC-3′. 11β-hydroxysteroid de-hydrogenase (HSD) 1 sense 5′-GTCCTTGGCCTCATAGACACAG-3′ antisense 5′-GGAGTCAAAGGCGATTTGTCAT. 11β-HSD2 sense 5′-GTTAACAACGCTGGCCTCAATATC-3′ anti-sense 5′-CAACGGTCACAATACGTCCCCTC-3′. GRα sense 5′-AAAGAGCTAGGAAAAGCCATTGTC-3′ antisense 5′-TCAGCTAACATCTCTGGGAATTCA-3′. GRβ sense 5′-AAAGAGCTAGGAAAAGCCATTGTC-3′ antisense 5′-CTGTCTTTGGGCTTTTGAGATAGG-3′ ERα sense 5′-CCGTGTGCAATGACTATGCC-3′ antisense 5′-GTGCTTCAACATTCTCCCTCCTC-3′. ERβ sense 5′-CTGTGATGAACTACAGTGTTCCC-3′ antisense 5′-CACATTTGGGCTTGCAGTCTG-3′. Androgen receptor (AR) sense 5′-TGGGACCTTGGATGGAGAAC-3′ antisense 5′-CTGGTACTGTCCAAACGCATGT-3′.

The amplification cycles for 11β-HSD1, 11β-HSD2, GRα, GRβ, ERα, ERβ, AR, and β-actin were 95°C for 15 s, 58°C for 30 s and 72°C for 60 s; for RANKL and OPG were 95°C for 15 s, 60°C for 30 s and 72°C for 60 s. All primer sets yielded a single product of the correct size. Relative expression levels were normalized against β-actin.

### Statistical Analysis

Statistical analysis was performed with one-way analysis of variance, followed by the *post hoc* Tukey test, using GraphPad Prism 5.0 software. The data are represented as means ± SEM.

## Results

### Adrenal Steroids Modulate *B. abortus* Intracellular Replication in Osteoblast

**Figure 1:**
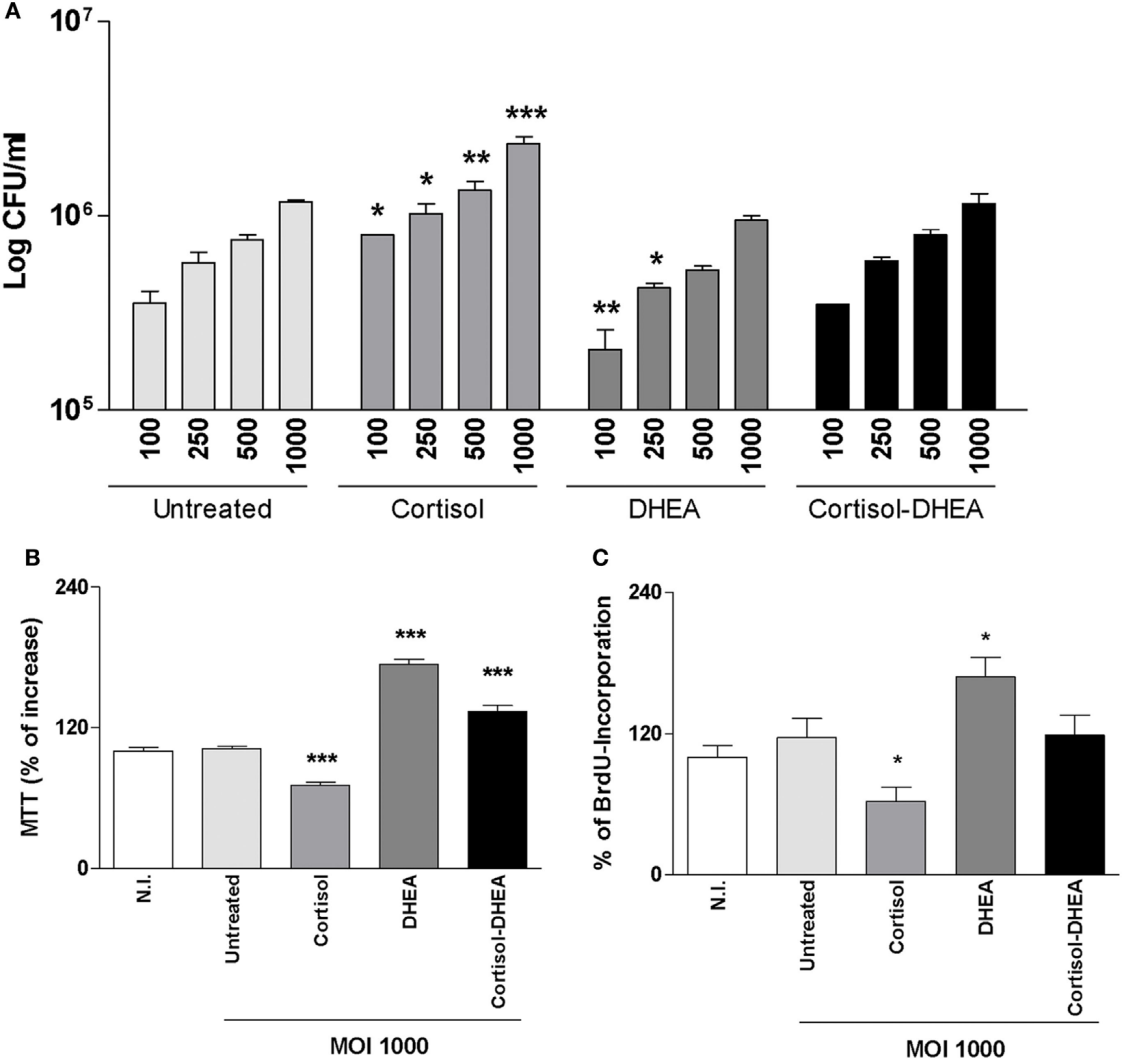
Adrenal steroids modulate intracellular replication of *Brucella abortus* and MC3T3-E1 proliferation. **(A)** After infection at different multiplicities of infection (MOI) (100, 250, 500, and 1,000) in the presence or not of cortisol (1 × 10^−6^ M), dehydroepiandrosterone (DHEA) (1 × 10^−8^ M), or cortisol plus DHEA (1 × 10^−6^ and 1 × 10^−8^ M, respectively), cells were incubated with antibiotics to kill extracellular bacteria. Cell lysates obtained at 24 h postinfection were plated onto agar to determine intracellular colony forming units (CFU). **(B,C)** After 48 h postinfection, cell proliferation was measured by MTT colorimetric assay **(B)** and ELISA bromodeoxyuridine (BrdU) **(C)**. Results were expressed as % of control non-infected (N.I.). Data are given as means ± SEM from at least four individual experiments. Significance of results for treated versus untreated cells: *P < 0.1; **P < 0.01; and ***P < 0.001.

Adrenal steroids do not only modulate the function of host cells but can also modulate bacterial intracellular replication, including *B. abortus* replication in monocytes/macrophages (11, 20, 21). We have previously demonstrated that human/mouse osteoblast cell lines and primary mouse osteoblast support *B. abortus* invasion and replication (7). Then, we decided to evaluate if cortisol and DHEA treatment could modify the capacity of *B. abortus* to replicate into osteoblast. *B. abortus* replicates in MC3T3-E1 osteoblasts. Cortisol significantly increased the capacity of *B. abortus* to replicate in osteoblasts with respect to untreated cells. By contrast, DHEA significantly decreased the intracellular *B. abortus* replication with respect to untreated cells at low MOI. When infection experiments were performed in the presence of both cortisol and DHEA, there were no differences in intracellular bacterial survival with respect to untreated cells (Figure 1A). In addition, we analyzed if cortisol and DHEA could have a direct effect on *B. abortus* replication. Our results indicated that, adrenal steroids treatment did not have a direct effect on bacterial replication, since CFU counts did not differ from untreated bacteria, measured at 2, 6, and 24 h posttreatment (not shown). Taken together these results indicate that cortisol treatment increases intracellular replication and DHEA is able to reverse the effect of cortisol.

### Adrenal Steroids Modulate Apoptosis and Proliferation of Osteoblast Precursors during *B. abortus* Infection

Osteoblast differentiation *in vitro* and *in vivo* can be characterized by a first state of cell proliferation (22). To appraise osteoblast proliferation, cells were infected with *B. abortus* in the presence of cortisol, DHEA or both, and cell proliferation was evaluated by MTT colorimetric assay and BrdU incorporation at 48 h postinfection. *B. abortus* was not capable of inducing osteoblast proliferation *in vitro*. However, when infection experiments were performed in the presence of cortisol, osteoblast proliferation was inhibited respect to untreated cells. By contrast, the presence of DHEA during *B. abortus* infection induced osteoblast proliferation. When infection experiments were performed in the presence of both cortisol and DHEA, our results indicate that DHEA could reverse the effect of cortisol on osteoblast proliferation (Figures 1B,C).

**Figure 2:**
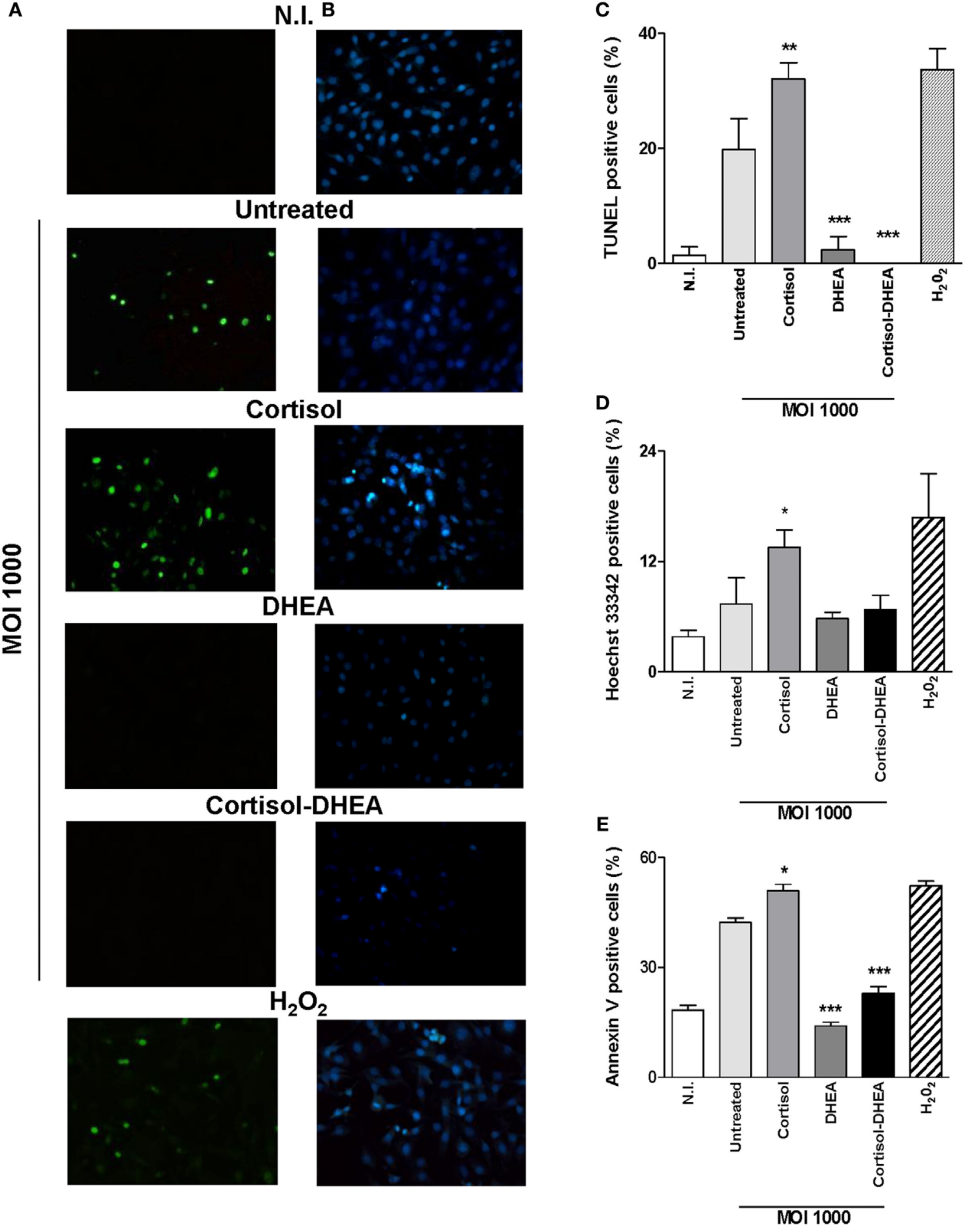
Dehydroepiandrosterone (DHEA) reverses the apoptotic effect on MC3T3-E1 cells induced by *Brucella abortus* infection. After infection at multiplicities of infection (MOI) 1,000 in the presence or not of cortisol (1 × 10^−6^ M), DHEA (1 × 10^−8^ M), or cortisol plus DHEA (1 × 10^−6^ and 1 × 10^−8^ M, respectively), cells were incubated with antibiotics to kill extracellular bacteria. Apoptosis was evaluated by terminal deoxynucleotidyltransferase-mediated dUTP-biotin nick end labeling (TUNEL), Hoechst 33342, and annexin V/propidium iodide (PI) techniques. Fluorescence microscopy analysis of apoptotic cells by TUNEL **(A,C)** and Hoechst 33342 **(B,D)**. Flow cytometry analysis of apoptotic cells by annexin V/PI **(E)**. H_2_O_2_ (200 µM) was used as a positive control. Abbreviation: N.I., non-infected. Data are given as means ± SEM from at least three individual experiments. Significance of results for treated versus untreated cells: *P < 0.1; **P < 0.01; and ***P < 0.001.

Osteoblast apoptosis is known to be involved in bone loss and results in the elimination of the cells responsible for matrix deposition (23). *B. abortus* infection induces apoptosis of osteoblast (13). Cortisol is not able to induce apoptosis in uninfected osteoblast (24). However, it has been demonstrated an inhibitory effect of apoptosis by DHEA treatment (25). Therefore, we aimed to investigate if cortisol and DHEA could modulate osteoblast apoptosis upon infection with *B. abortus*. Cortisol treatment significantly increased osteoblast apoptosis induced by *B. abortus* infection as determined after 24 h postinfection by annexin V–propidium iodide (PI) and analyzed by flow cytometry; and confirmed by TUNEL and Hoechst 33342 staining (Figure 2). By contrast, DHEA inhibits osteoblast apoptosis upon *B. abortus* infection. Moreover, DHEA could reverse the increasing apoptotic effect observed with cortisol; since when experiments were performed with both cortisol and DHEA, DHEA was also able to reverse the apoptotic effect of *B. abortus* infection in the presence of cortisol treatment (Figure 2). Culture of uninfected osteoblast with cortisol and DHEA resulted in no changes in the number of apoptotic cells respect to untreated cells (not shown).

Taken together, these results indicate that DHEA can reverse the effect of *B. abortus* infection on osteoblasts even when infection is performed in the presence of cortisol.

### Adrenal Steroids Modulate Osteoblast Differentiation during *B. abortus* Infection

**Figure 3:**
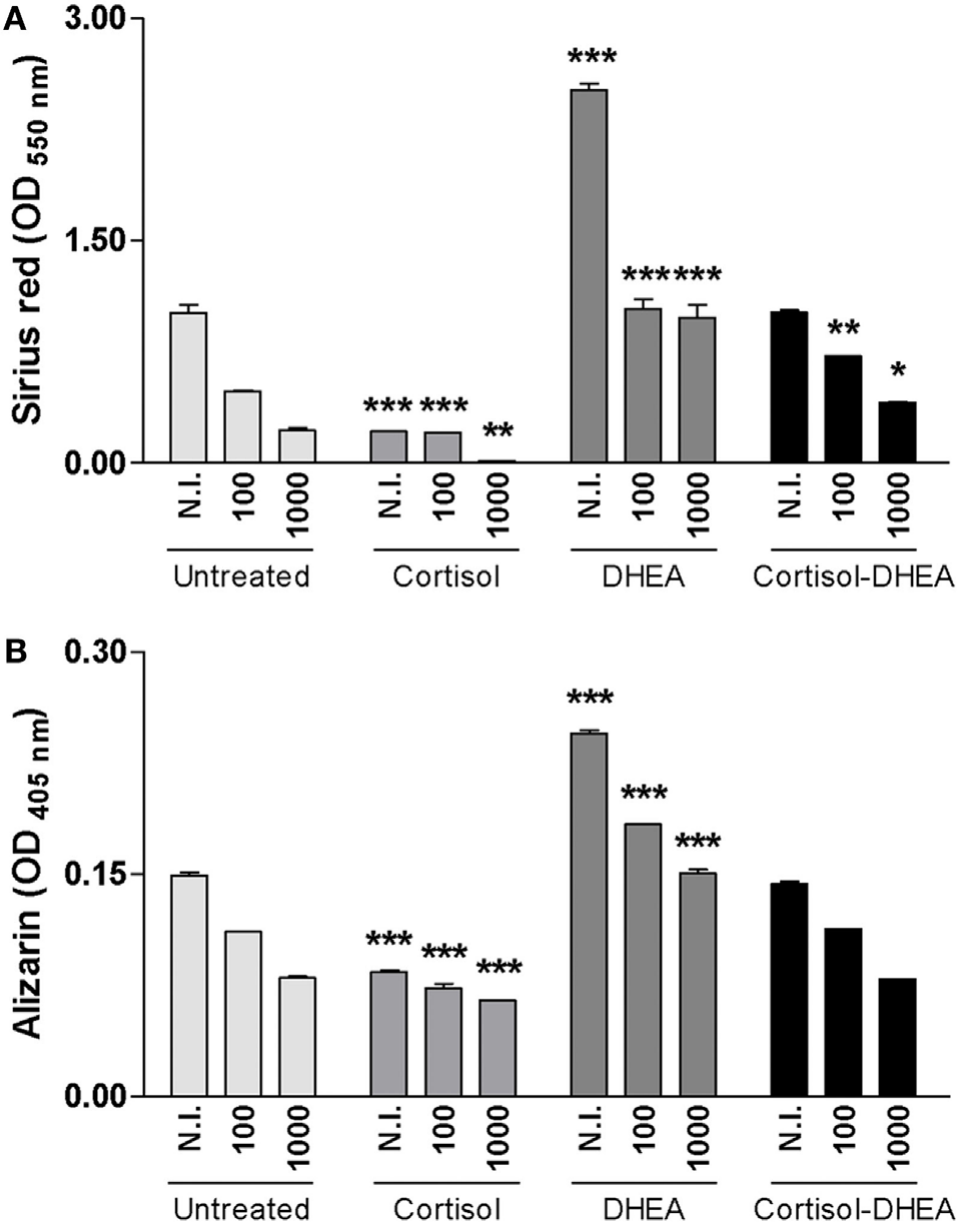
Adrenal steroids modulate osteoblast differentiation during *Brucella abortus* infection. After infection at multiplicities of infection 100 and 1,000 in the presence or not of cortisol (1 × 10^−6^ M), dehydroepiandrosterone (DHEA) (1 × 10^−8^ M), or cortisol plus DHEA (1 × 10^−6^ and 1 × 10^−8^ M, respectively), cells were incubated with antibiotics to kill extracellular bacteria. Collagen deposition was assessed by quantification of Sirius red staining at 7 days postinfection **(A)**. Calcium deposition was revealed by quantification of alizarin red S staining at 7 days postinfection **(B)**. Abbreviation: N.I., non-infected. Data are given as means ± SEM from at least four individual experiments. Significance of results for treated versus untreated cells: *P < 0.1; **P < 0.01; and ***P < 0.001.

Osteoblast differentiation *in vivo* and also *in vitro* is characterized by the deposition and mineralization of bone matrix (26). Glucocorticoids clearly cause detrimental effects on bone physiology (27), By contrast, DHEA has anabolic effects on bone (28). We have previously demonstrated that *B. abortus* infection inhibits osteoblast differentiation and bone formation through inhibiting organic and mineral matrix deposition (13). We next investigated the role of adrenal steroids on osteoblast differentiation in the context of *B. abortus* infection. To this end, calcium rich deposits and collagen deposition on osteoblast were determined by alizarin red S and Sirius red staining, respectively. Cortisol increased the inhibition of mineral and organic matrix deposition in *B. abortus*-infected osteoblast cells. By contrast, DHEA resulted in significant recovery of the inhibited collagen and calcium deposition mediated by *B. abortus* infection (Figure 3). When the two hormones were administrated together, the effect was similar to that of infected and untreated cells (Figure 3). Taken together, these results indicate that cortisol treatment increases the inhibition of osteoblast organic and mineral matrix deposition in *B. abortus*-infected osteoblast, by contrast, DHEA treatment is able to reverse the inhibitory effect of *B. abortus* infection on osteoblast function.

### Cortisol Inhibits RANKL, Chemokine, and MMP-2 Expression Induced by *B. abortus* Infection

**Figure 4:**
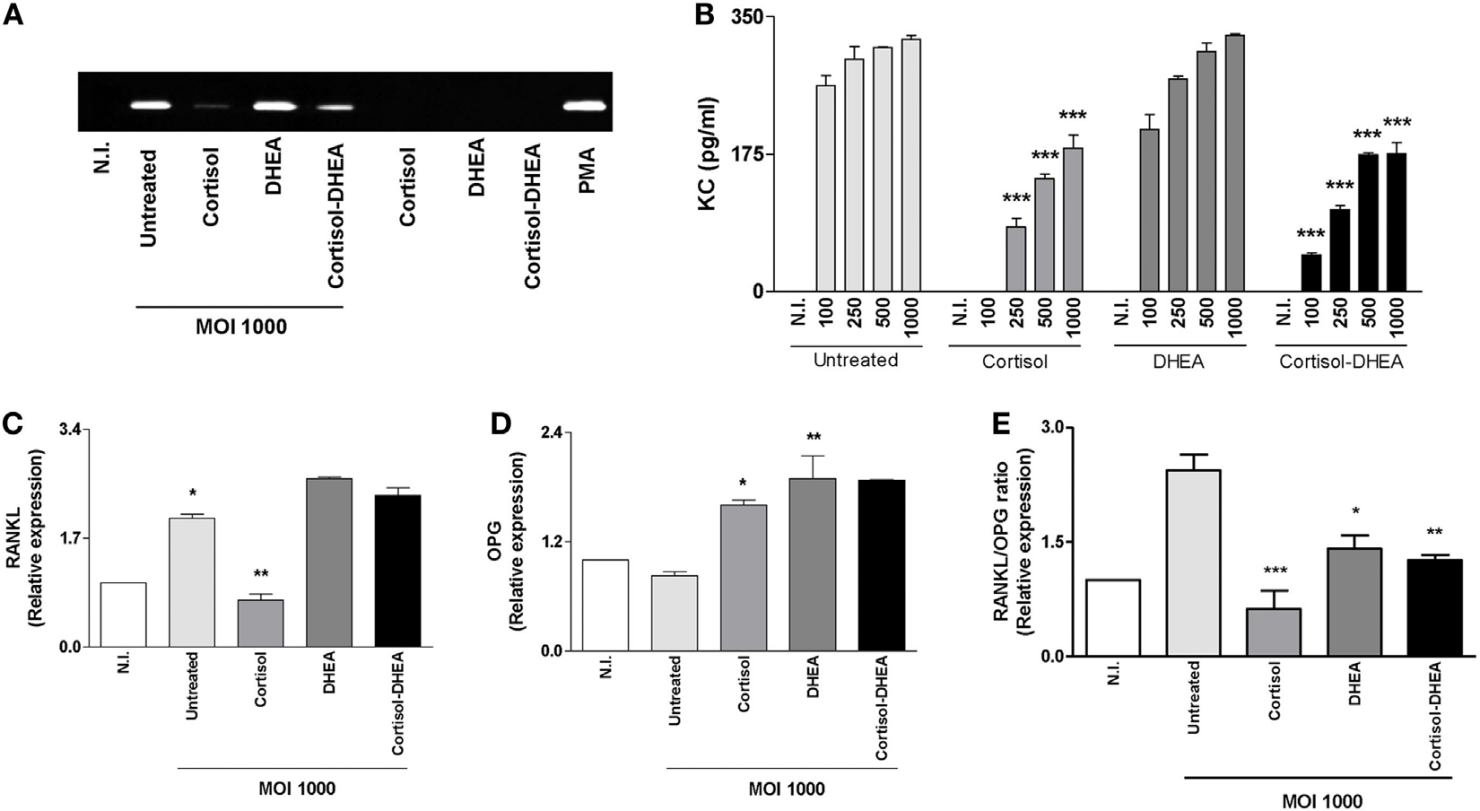
Cortisol inhibits receptor activator of nuclear factor-κB ligand (RANKL), keratinocyte chemoattractant (KC), and matrix metalloproteinase (MMP)-2 expression induced by *Brucella abortus* infection. After infection at indicated multiplicities of infection (MOI) in the presence or not of cortisol (1 × 10^−6^ M), dehydroepiandrosterone (DHEA) (1 × 10^−8^ M), or cortisol plus DHEA (1 × 10^−6^ and 1 × 10^−8^ M, respectively), cells were incubated with antibiotics to kill extracellular bacteria during 24 h. MMP-2 production by *B. abortus*-infected MC3T3-E1 cells was determined in supernatants by zymography **(A)**. Abbreviations: PMA: phorbol myristate acetate; N.I., non-infected. ELISA determination of the chemokine KC **(B)**, RANKL **(C)**, and osteoprotegerin (OPG) **(D)** expression determined by RT-qPCR. RANKL/OPG ratio **(E)**. Data are given as means ± SEM from at least four individual experiments. Significance of results for treated versus untreated cells: *P < 0.1; **P < 0.01; and ***P < 0.001.

Receptor activator of nuclear factor-κB ligand is a key molecule implicated in bone remodeling under physiological conditions (29). OPG is RANKL’s decoy receptor, reducing RANKL–RANK interactions and thus inhibits osteoclastogenesis. RANKL/OPG ratio is upregulated in pathological conditions causing bone resorption. Chemokines and MMPs play an important role in facilitating the migration of innate inflammatory cells (30), in addition increased levels of MMP activity cause tissue damage (3). As we have previously demonstrated, osteoblast infected with *B. abortus* induces RANKL, KC, and MMP-2 expression (19). Thus, we decided to investigate the ability of adrenal steroids to modulate the expression of RANKL/OPG, KC, and MMP-2 by osteoblasts upon *B. abortus* infection. Accordingly with our previous findings *B. abortus* infection induced an increase in RANKL, KC, and MMP-2 expression. When osteoblasts were infected with *B. abortus* in the presence of cortisol, these cells secreted significantly lower quantities of KC and MMP-2 and the RANKL/OPG ratio was significantly reduced respect to untreated cells. By contrast, treatment with DHEA reversed the inhibitory effect induced by cortisol treatment on the RANKL/OPG ratio (Figure 4). These results indicate that cortisol reduces the expression of proinflammatory mediators induced by *B. abortus* infection in osteoblasts and DHEA cannot reverse this effect.

### Glucocorticoid Receptor (GR) Expression Is Modulated by Adrenal Steroids during *B. abortus* Infection

**Figure 5:**
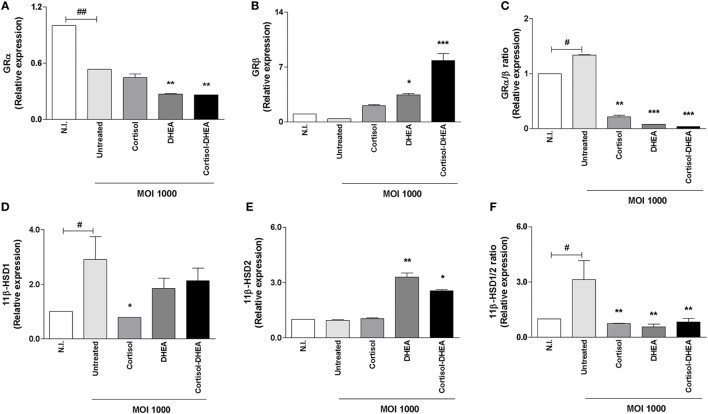
Adrenals steroids modulate glucocorticoid receptor (GR) and isoenzymes 11β-hydroxysteroid dehydrogenase (HSD) during *Brucella abortus* infection. GRα, GRβ, 11β-HSD1, and 11β-HSD2 expression were determined by RT-qPCR in MC3T3-E1 cells infected by *B. abortus* at multiplicities of infection (MOI) 1,000 in the presence or not of cortisol (1 × 10^−6^ M), dehydroepiandrosterone (DHEA) (1 × 10^−8^ M), or cortisol plus DHEA (1 × 10^−6^ and 1 × 10^−8^ M, respectively), for 24 h. GRα **(A)**, GRβ **(B)**, GRα/β ratio **(C)**, 11β-HSD1 **(D)**, 11β-HSD2 **(E)** and 11β-HSD1/2 ratio **(F)**. Abbreviation: N.I., non-infected. Data are given as means ± SEM from at least four individual experiments. Significance of results for treated versus untreated cells: *P < 0.1; **P < 0.01; and ***P < 0.001. Significance of results for uninfected versus untreated and infected: ^#^*P* < 0.05 and ^##^*P* < 0.01.

Cortisol binding to the GR regulates the expression of a wide array of target genes. The capacity of cells to respond to cortisol depends at least in part of levels of circulating cortisol and the activity of GR. Thus, experiments were conducted to establish if *B. abortus* infection could modulate GR expression and to determine if this phenomenon could be modulated by adrenal steroids treatment during the infection. *B. abortus* infection inhibits GRα, and this inhibition could not be reversed by cortisol or DHEA treatment. In addition, DHEA significantly inhibits the expression of GRα during *B. abortus* infection respect to untreated and infected cells (Figure 5A). When we analyzed the effect of *B. abortus* infection on GRβ expression, our result indicated that *B. abortus* did not induce changes in the expression of GRβ (Figure 5B). The presence of DHEA during *B. abortus* infection induced the expression of GRβ in osteoblasts, while cortisol had no effect. When infection experiments were performed in the presence of both cortisol and DHEA simultaneously, we observed a significant increase of GRβ expression over that seem in *B. abortus*-infected and untreated cells (Figures 5A,B).

GRβ lacks the ability to bind glucocorticoids, and it seems to function as an inhibitor of GRα-mediated transcriptional activation through the formation of GRα/GRβ heterodimers (31). In this context, our results indicate that *B. abortus* infection induces an increase in GRα/β ratio, but treatment with cortisol, DHEA and both cortisol and DHEA simultaneously is able to reverse these phenomena (Figure 5C).

### Adrenal Steroids Modulate Isoenzymes 11β-HSD during *B. abortus* Infection

The capacity of cells to respond to cortisol not only depends on GR expression but also it is dependent on its intracellular bioavailability (32). Levels of intracellular cortisol are dependent on the activity of the isoenzymes 11β-hydroxysteroid-dehydrogenase type 1 (11β-HSD1) and type 2 (11β-HSD2) that catalyze the interconversion of active cortisol to the inactive cortisone. Alterations in the expression of these isoenzymes in bone cells have been associated with bone loss (33). Then, experiments were conducted to determine if *B. abortus* infection could modulate 11β-HSD1 and 11β-HSD2 expression. *B. abortus* infection induced 11β-HSD1 expression (Figure 5D). When infection experiments were performed in the presence of cortisol, this hormone inhibited 11β-HSD1 expression induced by *B. abortus* infection. By contrast, DHEA treatment had no effect on the expression of 11β-HSD1. When infection experiments were performed in the presence of both cortisol and DHEA simultaneously, we observed that DHEA could reverse the inhibitory effect of cortisol (Figure 5D).

By contrast, *B. abortus* infection did not induce changes in the expression of 11β-HSD2 (Figure 5E). The presence of DHEA during *B. abortus* infection induced the expression of 11β-HSD2 in osteoblast. Cortisol treatment had no effect on the expression of this molecule when it was added during *B. abortus* infection. When infection experiments were performed in the presence of both cortisol and DHEA simultaneously, we observed a significant increase of 11β-HSD2 expression over that seen in *B. abortus*-infected untreated cells or cells treated with cortisol alone (Figure 5E). When we analyzed 11β-HSD1/2 ratio, our results indicated that *B. abortus* infection induced an increase in 11β-HSD1/2 ratio but cortisol and DHEA treatment was able to reverse this effect (Figure 5F).

### ERK1/2 Is Involved in the Modulation of Osteoblast Function Induced by DHEA during *B. abortus* Infection

**Figure 6:**
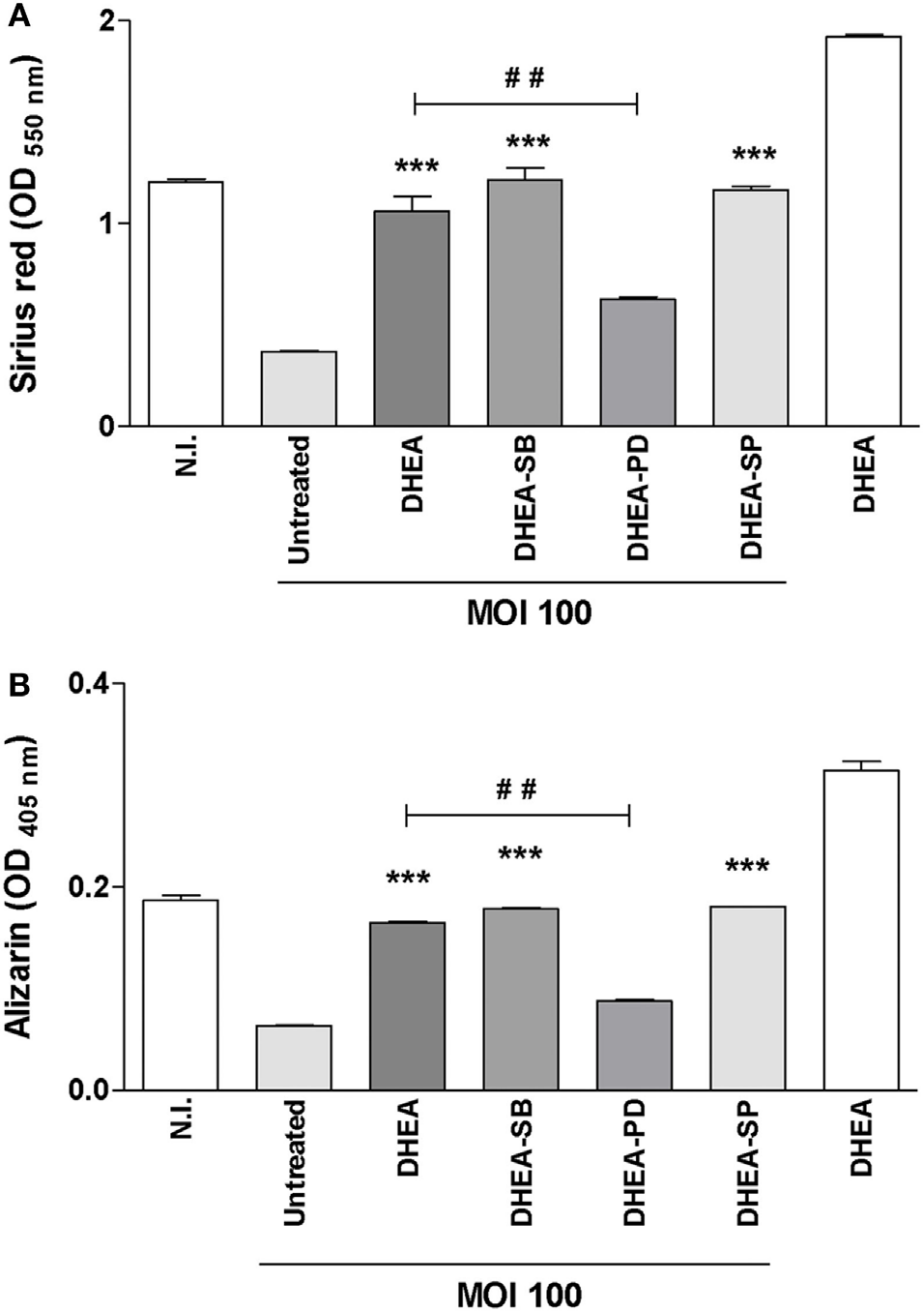
ERK1/2 is involved in the modulation of osteoblast differentiation induced by dehydroepiandrosterone (DHEA) during *Brucella abortus* infection. MC3T3-E1 cells infected by *B. abortus* at multiplicities of infection (MOI) 1,000 in the presence or not of cortisol (1 × 10^−6^ M), DHEA (1 × 10^−8^ M), or cortisol plus DHEA (1 × 10^−6^ and 1 × 10^−8^ M, respectively) and inhibitors of MAPK pathways (PD98059: ERK1/2 inhibitor; SB203580: p38 inhibitor; and SP600125: JNK1/2 inhibitor). Collagen deposition was assessed by quantification of Sirius red staining at 7 days postinfection **(A)**. Calcium deposition was revealed by quantification of alizarin red S staining at 7 days postinfection **(B)**. Abbreviation: N.I., non-infected. Data are given as means ± SEM from at least four individual experiments. Significance of results for treated versus untreated cells: ***P < 0.001. Significance of results for DHEA versus DHEA plus ERK1/2 inhibitor (DHEA-PD): ^#^*P* < 0.01.

ERK is pivotal for differentiation and cell function in human osteoblastic cells (34, 35). In addition, it has been described that DHEA modulates osteoblast function in a way that involves mainly the ERK pathway (25, 36). Thus, to gain insight into the signaling pathways involved in the modulation of osteoblast differentiation we investigated the role of ERK1/2, p38, and JNK MAPK in the modulation of matrix deposition mediated by *B. abortus* infection in the presence or absence of DHEA. To this end, osteoblast cells were infected in the presence of DHEA and the specific inhibitors of MAPK signaling pathways, PD98059, SB203580, and SP600125, which inhibit ERK1/2, p38, and JNK1/2, respectively. The ERK1/2 inhibitor was able to reverse the effect of DHEA on mineral and organic matrix deposition during *B. abortus* infection (Figure 6). Conversely, inhibition of p38 or JNK1/2 had no effect. These results indicate that during *B. abortus* infection and in the context DHEA treatment the modulation of organic and mineral matrix deposition depends on ERK1/2 pathway.

### Adrenal Steroids Modulate ER and AR during *B. abortus* Infection

**Figure 7:**
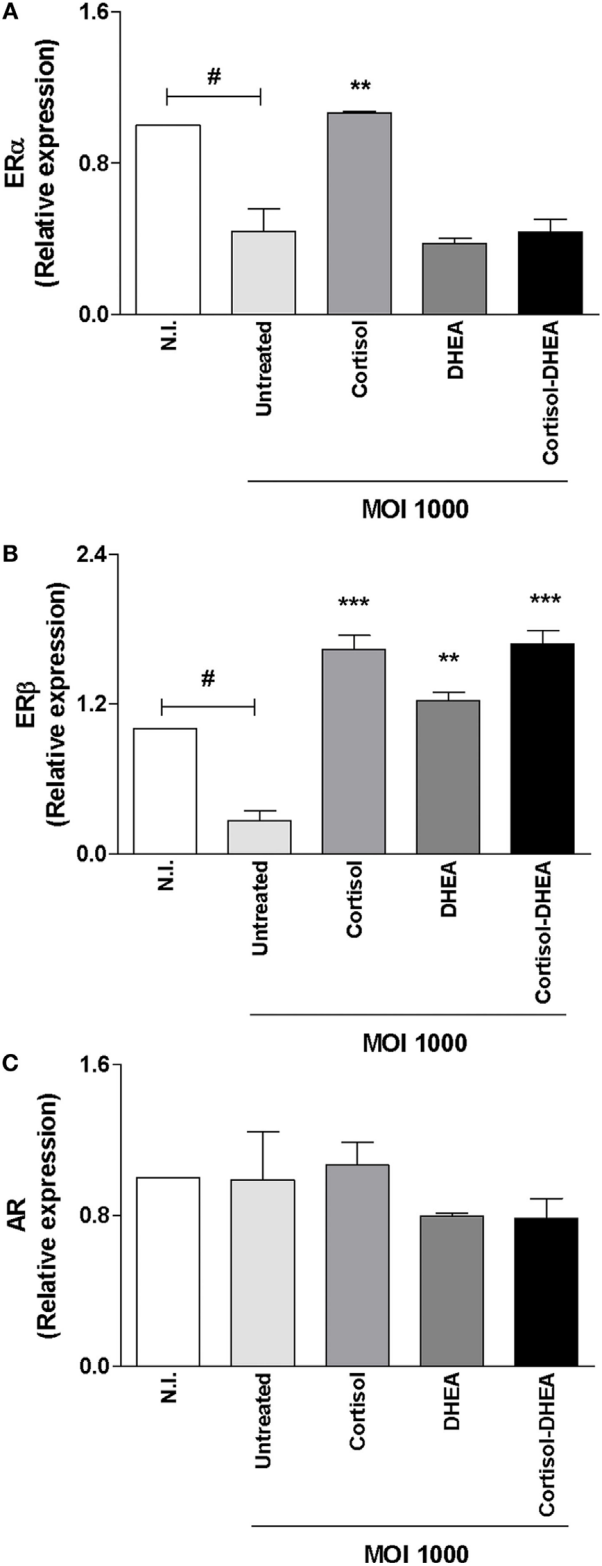
Adrenal steroids modulate estrogen receptor (ER) and androgen receptor (AR) during *Brucella abortus* infection. ER and AR expression were determined by RT-qPCR in MC3T3-E1 cells infected by *B. abortus* at multiplicities of infection (MOI) 1,000 in the presence or not of cortisol (1 × 10^−6^ M), dehydroepiandrosterone (DHEA) (1 × 10^−8^ M), or cortisol plus DHEA (1 × 10^−6^ and 1 × 10^−8^ M, respectively), for 24 h. ERα **(A)**, ERβ **(B)**, and AR **(C)**. Abbreviation: N.I., non-infected. Data are given as means ± SEM from at least four individual experiments. Significance of results for treated versus untreated cells: **P < 0.01 and ***P < 0.001. Significance of results for uninfected versus untreated and infected: ^#^*P* < 0.05.

It has been reported that ER and AR are involved in the action of DHEA (37, 38). Then, experiments were conducted to determine the expression of ER and AR during *B. abortus* infection and their modulation by cortisol and DHEA. *B. abortus* infection inhibits ERα expression, and this phenomenon was reversed when infection experiments were performed in the presence of cortisol (Figure 7A). By contrast, when infection experiments were performed in the presence of DHEA, our results indicate that DHEA could not reverse the inhibitory effect of *B. abortus* infection on ERα expression. When cells were infected in the presence of both DHEA and cortisol, ERα expression was similar to that produced by *B. abortus*-infected cells (Figure 7A) *B. abortus* infection inhibits ERβ expression, and cortisol and DHEA reversed the inhibitory effect of *B. abortus* infection on ERβ expression (Figure 7B). AR expression was not modulated by *B. abortus* infection or adrenal steroids treatment (Figure 7C).

Although the ability of AR and ER to mediate DHEA signaling was described in different cell types (37, 38); it has been reported that DHEA seems to act on osteoblast through the MAPK signaling pathway involving ERK1/2 in a way that is mainly modulated by ERβ instead of AR. In concordance, DHEA could reverse the inhibitory effect of *B. abortus* infection on ERβ expression in osteoblast (Figure 7B).

### DHEA Reversed the Inhibitory Effect of *B. abortus* in Matrix Deposition *via* ER

**Figure 8:**
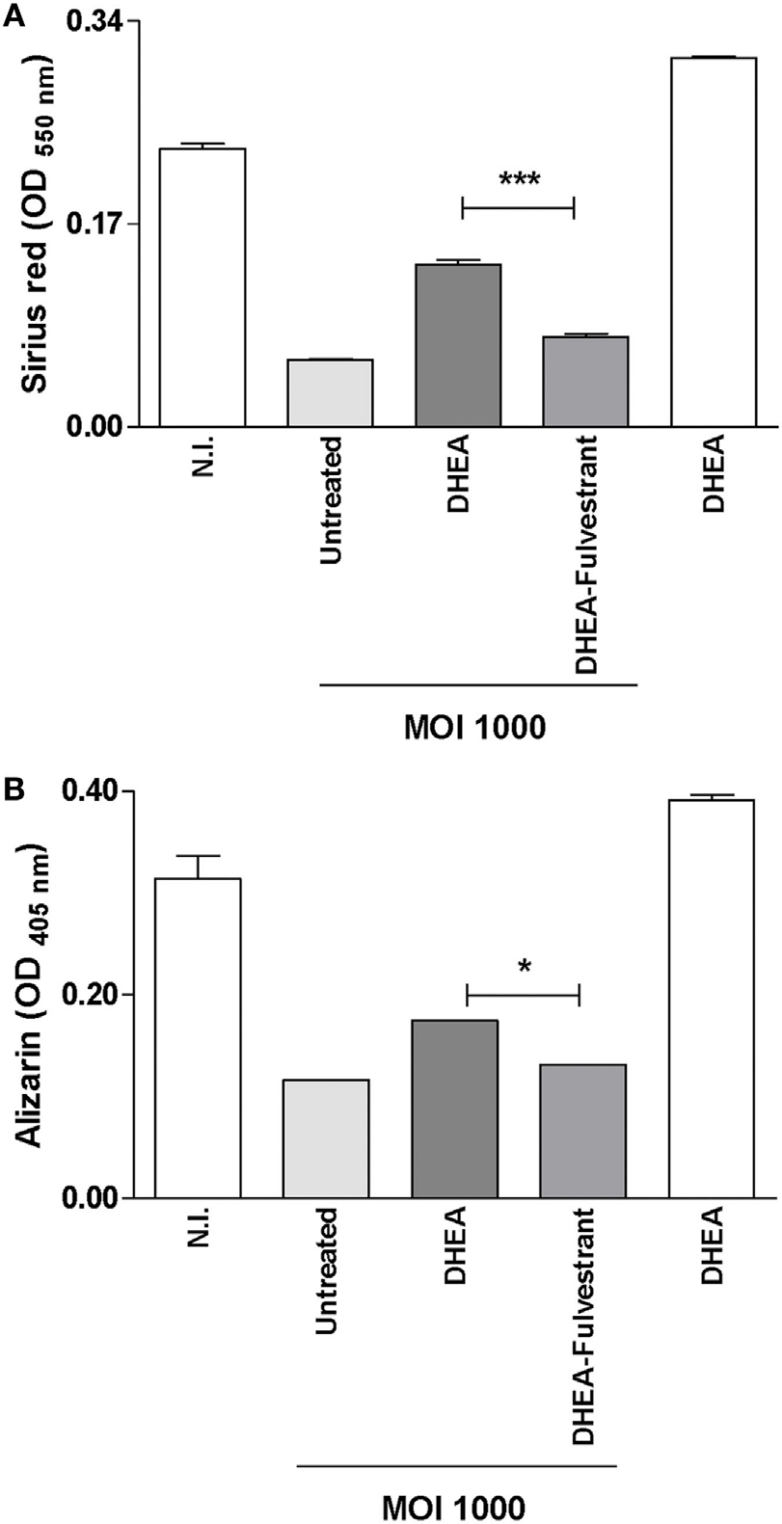
Dehydroepiandrosterone (DHEA) reversed the inhibitory effect of *Brucella abortus* in matrix deposition *via* estrogen receptor (ER). MC3T3-E1 cells infected by *B. abortus* at multiplicities of infection (MOI) 1,000 in the presence or not of cortisol (1 × 10^−6^ M), DHEA (1 × 10^−8^ M), or cortisol plus DHEA (1 × 10^−6^ and 1 × 10^−8^ M, respectively) and inhibitor of ER (fulvestrant). Collagen deposition was assessed by quantification of Sirius red staining at 7 days postinfection **(A)**. Calcium deposition was revealed by quantification of alizarin red S staining at 7 days postinfection **(B)**. Abbreviation: N.I., non-infected. Data are given as means ± SEM from at least four individual experiments. Significance of results for treated versus untreated cells: *P < 0.1 and ***P < 0.001.

Studies were conducted to determine the role of ER in the reversion of the inhibitory effect on matrix deposition induced by DHEA during *B. abortus* infection in osteoblast. To this end, fulvestrant-mediated ER inhibition was employed to seek for a mechanistic role of ER in regulating DHEA-effects, on organic and mineral matrix deposition in *B. abortus*-infected osteoblast in the presence of DHEA. Fulvestrant was able to reverse the effect of DHEA in the induction of mineral and organic matrix deposition in *B. abortus-*infected osteoblast (Figure 8). Therefore, these results indicate that DHEA mediates effect on *B. abortus*-infected osteoblast mainly through ER receptor.

## Discussion

Although bone is a tissue resistant to infection, osteoarticular involvement is the most prevalent local form of brucellosis. Many of the molecular mechanisms of bone damage during *Brucella* infection have been recently described (6). In particular, *B. abortus* infection is able to affect osteoblast physiology, since we have previously demonstrated that infection induces osteoblast apoptosis and inhibits osteoblast differentiation and function. Then, infection of osteoblast contributes to bone damage through the inhibition of new matrix deposition and by inducing an increase of RANKL expression with concomitant osteoclastogenesis and bone matrix resorption (16).

In brucellosis, we and others have demonstrated that cortisol/DHEA ratio is elevated in infected patients with acute disease with respect to healthy individuals and patients with remission (10, 11). This increase could modulate bone responses and, in particular, osteoblast responses during osteoarticular localization of *Brucella* infection.

Our results indicated that cortisol treatment significantly increased *B. abortus* intracellular proliferation in osteoblasts, and this phenomenon was reversed when both cortisol and DHEA were administrated simultaneously. The impact of glucocorticoids on intracellular bacterial burden increase has been described previously in macrophages infected with *B. abortus, Salmonella typhimurium* and *Mycobacterium tuberculosis* (11, 20, 21). This indicates that cortisol could not only promote *B. abortus* infection through the inhibition of the immune response but also favors its intracellular multiplication. Interestingly, the number of intracellular bacteria was reduced when infections were performed in the presence of DHEA. Moreover, DHEA reversed the effect of cortisol, as observed in cells treated with cortisol and DHEA in conjunction. This result adds further support to the beneficial effect of DHEA that was previously reported in non-infectious bone disease (27). The beneficial effect of DHEA on osteoblasts during *B. abortus* infection was also highlighted when the apoptosis of osteoblasts was assessed. We have previously demonstrated that *B. abortus* infection induces osteoblast apoptosis (16). This phenomenon was increased by cortisol treatment. Cortisol is not able to induce apoptosis in uninfected osteoblast (24) By contrast, it has been described that other glucocorticoids could induce osteoblast apoptosis (39); however, the effect of glucocorticoid is dependent on the glucocorticoid type, concentration, and target cell, intracellular and extracellular milieu (40). This could explain at least in part, the increase of apoptosis observed in *B. abortus*-infected osteoblast in the presence of cortisol, when cortisol was unable to induce apoptosis in uninfected cells. By contrast, DHEA treatment could reverse the apoptotic effect in infected cells and also when infection experiments were performed in the presence of cortisol.

Tissue cortisol sensitivity not only depends on its availability but also on the relationship between both GR isoforms (GRα and GRβ) in the target cell. Also, the intracellular availability of cortisol depends on the expression of 11β-HSD1 and 11β-HSD2 enzymes that catalyze cortisol-cortisone interconversion. Our results indicates that *B. abortus* was able to increase GRα/β and 11β-HSD1/2 ratio in osteoblasts suggesting an augmented intracellular concentration of cortisol. This increase in GRα/β and 11β-HSD1/2 expression was also observed in mononuclear cells from patients infected with *M. tuberculosis* (41). DHEA treatment reduced GRα/β and 11β-HSD1/2 ratio in infected osteoblast. Thus, DHEA could offer a protective role in *B. abortus*-infected osteoblast not only through the increase of GRβ expression that lacks the ability to bind glucocorticoids but also by increasing 11β-HSD2 expression with the concomitant reduction of the intracellular availability of cortisol.

Adrenal hormones modulate osteoblast differentiation and function (27). However, until now, their function has not been investigated in the context of bacterial infection. *B. abortus* infection inhibits matrix deposition by osteoblast (16), and this response was reversed by DHEA treatment through a mechanism that is dependent on ER and ERK1/2 MAPK. Until now, DHEA effect on osteoblast function has been studied in the context of non-infectious bone diseases (27, 42). Then, these results extend the role of DHEA to bone disease caused by bacterial infections.

It has been demonstrated that DHEA and cortisol can modulate cytokines, chemokines, and MMPs expression in the context of bacterial infection, including *B. abortus*, in others cells types (11, 21, 43). Adrenal steroids could not only contribute to bone damage by acting directly on bone cells but also they could induce chemokine secretion with the concomitant attraction of immune cells that could create a proinflammatory microenvironment leading to bone resorption. We previously have found that *B. abortus* infection induces the expression of RANKL, KC and MMP-2 (16). Here, we demonstrated that cortisol inhibits the production of these mediators in osteoblast during *B. abortus* infection and DHEA treatment had no effect on KC and MMP-2 expression.

Finally, this study constitutes the first analysis on the adrenal steroids modulation of osteoblast function in a context of a bacterial infection. This study provides an initial background to determine the role of DHEA in reducing the bone damage during *Brucella* infection, indicating that DHEA could modulate many osteoblast functions. In this context, antibiotic therapy with supplementation with DHEA or derivatives can be considered as a new possible treatment that reduces the bone damage that occurs during osteoarticular localization of brucellosis.

## Author Contributions

MG, AV, PB, and MD conceived and designed the experiments. MG, AV, and PB performed the experiments. MG and MD analyzed the data. MD and GG wrote the paper. AM and GC purchased reagents and prepared equipments. All the authors reviewed the manuscript.

## Conflict of Interest Statement

The authors declare that the research was conducted in the absence of any commercial or financial relationships that could be construed as a potential conflict of interest.

## References

[1] Turan H Serefhanoglu K Karadeli E Togan T Arslan H. Osteoarticular involvement among 202 brucellosis cases identified in Central Anatolia region of Turkey. Intern Med (2011) 50(5):421–8. 10.2169/internalmedicine.50.4700 21372451

[2] Aydin M Fuat Yapar A Savas L Reyhan M Pourbagher A Turunc TY Scintigraphic findings in osteoarticular brucellosis. Nucl Med Commun (2005) 26(7):639–47. 10.1097/01.mnm.0000167651.52724.68 15942485

[3] Gotuzzo E Alarcon GS Bocanegra TS Carrillo C Guerra JC Rolando I Articular involvement in human brucellosis: a retrospective analysis of 304 cases. Semin Arthritis Rheum (1982) 12(2):245–55. 10.1016/0049-0172(82)90064-6 6101216

[4] Madkour MM Osteoarticular brucellosis. 2nd ed In: Madkour MM, editor. Madkour’s Brucellosis. Berlin, Germany: Springer-Verlag (2001). p. 74–84.

[5] Pourbagher A Pourbagher MA Savas L Turunc T Demiroglu YZ Erol I Epidemiologic, clinical, and imaging findings in brucellosis patients with osteoarticular involvement. AJR Am J Roentgenol (2006) 187(4):873–80. 10.2214/AJR.05.1088 16985128

[6] Giambartolomei GH Arriola Benitez PC Delpino MV. *Brucella* and osteoarticular cell activation: partners in crime. Front Microbiol (2017) 8:256. 10.3389/fmicb.2017.00256 28265268 PMC5316522

[7] Delpino MV Fossati CA Baldi PC. Proinflammatory response of human osteoblastic cell lines and osteoblast-monocyte interaction upon infection with *Brucella* spp. Infect Immun (2009) 77(3):984–95. 10.1128/IAI.01259-08 19103778 PMC2643642

[8] Scian R Barrionuevo P Giambartolomei GH Fossati CA Baldi PC Delpino MV. Granulocyte-macrophage colony-stimulating factor- and tumor necrosis factor alpha-mediated matrix metalloproteinase production by human osteoblasts and monocytes after infection with *Brucella abortus*. Infect Immun (2011) 79(1):192–202. 10.1128/IAI.00934-10 20956574 PMC3019911

[9] Besedovsky HO del Rey A Immune-neuro-endocrine interactions: facts and hypotheses. Endocr Rev (1996) 17(1):64–102. 10.1210/edrv-17-1-64 8641224

[10] Yildiz O Gokce C Alp E Durak AC Aygen B Kelestimur F Investigation of the hypothalamo-pituitary-adrenal axis and changes in the size of adrenal glands in acute brucellosis. Endocr J (2005) 52(2):183–8. 10.1507/endocrj.52.183 15863945

[11] Gentilini MV Velasquez LN Barrionuevo P Arriola Benitez PC Giambartolomei GH Delpino MV. Adrenal steroids modulate the immune response during *Brucella abortus* infection by a mechanism that depends on the regulation of cytokine production. Infect Immun (2015) 83(5):1973–82. 10.1128/IAI.03090-14 25733519 PMC4399066

[12] Gorvel JP Moreno E. *Brucella* intracellular life: from invasion to intracellular replication. Vet Microbiol (2002) 90(1–4):281–97. 10.1016/S0378-1135(02)00214-6 12414149

[13] Delpino MV Barrionuevo P Macedo GC Oliveira SC Genaro SD Scian R Macrophage-elicited osteoclastogenesis in response to *Brucella abortus* infection requires TLR2/MyD88-dependent TNF-alpha production. J Leukoc Biol (2012) 91(2):285–98. 10.1189/jlb.04111185 22075930

[14] Hazeldine J Arlt W Lord JM. Dehydroepiandrosterone as a regulator of immune cell function. J Steroid Biochem Mol Biol (2010) 120(2–3):127–36. 10.1016/j.jsbmb.2009.12.016 20060904

[15] Lai WC Zhou M Shankavaram U Peng G Wahl LM. Differential regulation of lipopolysaccharide-induced monocyte matrix metalloproteinase (MMP)-1 and MMP-9 by p38 and extracellular signal-regulated kinase 1/2 mitogen-activated protein kinases. J Immunol (2003) 170(12):6244–9. 10.4049/jimmunol.170.12.6244 12794156

[16] Scian R Barrionuevo P Fossati CA Giambartolomei GH Delpino MV. *Brucella abortus* invasion of osteoblasts inhibits bone formation. Infect Immun (2012) 80(7):2333–45. 10.1128/IAI.00208-12 22547546 PMC3416452

[17] Bhattacharyya RS Krishnan AV Swami S Feldman D. Fulvestrant (ICI 182,780) down-regulates androgen receptor expression and diminishes androgenic responses in LNCaP human prostate cancer cells. Mol Cancer Ther (2006) 5(6):1539–49. 10.1158/1535-7163.MCT-06-0065 16818513

[18] Hibbs MS Hasty KA Seyer JM Kang AH Mainardi CL. Biochemical and immunological characterization of the secreted forms of human neutrophil gelatinase. J Biol Chem (1985) 260(4):2493–500. 2982822

[19] Scian R Barrionuevo P Giambartolomei GH De Simone EA Vanzulli SI Fossati CA Potential role of fibroblast-like synoviocytes in joint damage induced by *Brucella abortus* infection through production and induction of matrix metalloproteinases. Infect Immun (2011) 79(9):3619–32. 10.1128/IAI.05408-11 21730088 PMC3165475

[20] Verbrugghe E Boyen F Van Parys A Van Deun K Croubels S Thompson A Stress induced *Salmonella Typhimurium* recrudescence in pigs coincides with cortisol induced increased intracellular proliferation in macrophages. Vet Res (2011) 42:118. 10.1186/1297-9716-42-118 22151081 PMC3256119

[21] Bongiovanni B Mata-Espinosa D D’Attilio L Leon-Contreras JC Marquez-Velasco R Bottasso O Effect of cortisol and/or DHEA on THP1-derived macrophages infected with *Mycobacterium tuberculosis*. Tuberculosis (Edinb) (2015) 95(5):562–9. 10.1016/j.tube.2015.05.011 26099547

[22] Rutkovskiy A Stenslokken KO Vaage IJ. Osteoblast differentiation at a glance. Med Sci Monit Basic Res (2016) 22:95–106. 10.12659/MSMBR.901142 27667570 PMC5040224

[23] Hughes DE Boyce BF Apoptosis in bone physiology and disease. Mol Pathol (1997) 50(3):132–7. 10.1136/mp.50.3.132 9292147 PMC379607

[24] Pereira RM Delany AM Canalis E. Cortisol inhibits the differentiation and apoptosis of osteoblasts in culture. Bone (2001) 28(5):484–90. 10.1016/S8756-3282(01)00422-7 11344047

[25] Wang L Wang YD Wang WJ Zhu Y Li DJ. Dehydroepiandrosterone improves murine osteoblast growth and bone tissue morphometry via mitogen-activated protein kinase signaling pathway independent of either androgen receptor or estrogen receptor. J Mol Endocrinol (2007) 38(4):467–79. 10.1677/jme.1.02173 17446236

[26] Buckwalter JA Cooper RR. Bone structure and function. Instr Course Lect (1987) 36:27–48. 3325555

[27] Hardy R Cooper MS Adrenal gland and bone. Arch Biochem Biophys (2010) 503(1):137–45. 10.1016/j.abb.2010.06.007 20542010

[28] Liang X Glowacki J Hahne J Xie L LeBoff MS Zhou S. Dehydroepiandrosterone stimulation of osteoblastogenesis in human MSCs requires IGF-I signaling. J Cell Biochem (2016) 117(8):1769–74. 10.1002/jcb.25475 26682953

[29] Takayanagi H. The unexpected link between osteoclasts and the immune system. Adv Exp Med Biol (2010) 658:61–8. 10.1007/978-1-4419-1050-9_7 19950016

[30] Elkington PT O’Kane CM Friedland JS. The paradox of matrix metalloproteinases in infectious disease. Clin Exp Immunol (2005) 142(1):12–20. 10.1111/j.1365-2249.2005.02840.x 16178851 PMC1809491

[31] Oakley RH Cidlowski JA. Cellular processing of the glucocorticoid receptor gene and protein: new mechanisms for generating tissue-specific actions of glucocorticoids. J Biol Chem (2011) 286(5):3177–84. 10.1074/jbc.R110.179325 21149445 PMC3030321

[32] Oakley RH Cidlowski JA. The biology of the glucocorticoid receptor: new signaling mechanisms in health and disease. J Allergy Clin Immunol (2013) 132(5):1033–44. 10.1016/j.jaci.2013.09.007 24084075 PMC4084612

[33] Weinstein RS Wan C Liu Q Wang Y Almeida M O’Brien CA Endogenous glucocorticoids decrease skeletal angiogenesis, vascularity, hydration, and strength in aged mice. Aging Cell (2010) 9(2):147–61. 10.1111/j.1474-9726.2009.00545.x 20047574 PMC2858771

[34] Salasznyk RM Klees RF Hughlock MK Plopper GE. ERK signaling pathways regulate the osteogenic differentiation of human mesenchymal stem cells on collagen I and vitronectin. Cell Commun Adhes (2004) 11(5–6):137–53. 10.1080/15419060500242836 16194881

[35] Lai CF Chaudhary L Fausto A Halstead LR Ory DS Avioli LV Erk is essential for growth, differentiation, integrin expression, and cell function in human osteoblastic cells. J Biol Chem (2001) 276(17):14443–50. 10.1074/jbc.M010021200 11278600

[36] Liu D Iruthayanathan M Homan LL Wang Y Yang L Wang Y Dehydroepiandrosterone stimulates endothelial proliferation and angiogenesis through extracellular signal-regulated kinase 1/2-mediated mechanisms. Endocrinology (2008) 149(3):889–98. 10.1210/en.2007-1125 18079198 PMC2275364

[37] Engdahl C Lagerquist MK Stubelius A Andersson A Studer E Ohlsson C Role of androgen and estrogen receptors for the action of dehydroepiandrosterone (DHEA). Endocrinology (2014) 155(3):889–96. 10.1210/en.2013-1561 24424045

[38] Wang YD Tao MF Wang L Cheng WW Wan XP Selective regulation of osteoblastic OPG and RANKL by dehydroepiandrosterone through activation of the estrogen receptor beta-mediated MAPK signaling pathway. Horm Metab Res (2012) 44(7):494–500. 10.1055/s-0032-1311567 22556124

[39] Sato AY Tu X McAndrews KA Plotkin LI Bellido T. Prevention of glucocorticoid induced-apoptosis of osteoblasts and osteocytes by protecting against endoplasmic reticulum (ER) stress in vitro and in vivo in female mice. Bone (2015) 73:60–8. 10.1016/j.bone.2014.12.012 25532480 PMC4336847

[40] Schmidt S Rainer J Ploner C Presul E Riml S Kofler R. Glucocorticoid-induced apoptosis and glucocorticoid resistance: molecular mechanisms and clinical relevance. Cell Death Differ (2004) 11(Suppl 1):S45–55. 10.1038/sj.cdd.4401456 15243581

[41] D’Attilio L Diaz A Santucci N Bongiovanni B Gardenez W Marchesini M Levels of inflammatory cytokines, adrenal steroids, and mRNA for GRalpha, GRbeta and 11betaHSD1 in TB pleurisy. Tuberculosis (Edinb) (2013) 93(6):635–41. 10.1016/j.tube.2013.07.008 23988280

[42] Oberbeck R Kobbe P. Dehydroepiandrosterone (DHEA): a steroid with multiple effects. IS there any possible option in the treatment of critical illness? Curr Med Chem (2010) 17(11):1039–47. 10.2174/092986710790820570 20156161

[43] Perez AR Bottasso O Savino W. The impact of infectious diseases upon neuroendocrine circuits. Neuroimmunomodulation (2009) 16(2):96–105. 10.1159/000180264 19212129

